# State-Specific Prevalence of Adult Tobacco Product Use and Cigarette Smoking Cessation Behaviors, United States, 2018–2019

**DOI:** 10.5888/pcd20.230132

**Published:** 2023-11-16

**Authors:** Monica E. Cornelius, Teresa W. Wang, Ahmed Jamal, Caitlin G. Loretan, Gordon Willis, Bria Graham-Glover, Linda Neff

**Affiliations:** 1 Office on Smoking and Health, National Center for Chronic Disease Prevention and Health Promotion, Centers for Disease Control and Prevention, Atlanta, Georgia; 2 Behavioral Research Program, Division of Cancer Control and Population Sciences, National Cancer Institute, Rockville, Maryland; 3 Center for Tobacco Products, US Food and Drug Administration, Silver Spring, Maryland

## Abstract

**Introduction:**

Increasing quitting among people who smoke cigarettes is the quickest approach to reducing tobacco-related disease and death.

**Methods:**

We analyzed data from the 2018–2019 Tobacco Use Supplement to the Current Population Survey for 137,471 adult self-respondents from all 50 US states and the District of Columbia to estimate state-specific prevalence of current tobacco product use, interest in quitting smoking, past-year quit attempts, recent successful cessation (past-year quit lasting ≥6 months), receipt of advice to quit smoking from a medical doctor, and use of cessation medications and/or counseling to quit.

**Results:**

Prevalence of current any-tobacco use (use every day or some days) ranged from 10.2% in California to 29.0% in West Virginia. The percentage of adults who currently smoked cigarettes and were interested in quitting ranged from 68.2% in Alabama to 87.5% in Connecticut; made a past-year quit attempt ranged from 44.1% in Tennessee to 62.8% in Rhode Island; reported recent successful cessation ranged from 4.6% in West Virginia and Wisconsin to 10.8% in South Dakota; received advice to quit from a medical doctor ranged from 63.3% in Colorado to 86.9% in Rhode Island; and used medications and/or counseling to quit ranged from 25.5% in Nevada to 50.1% in Massachusetts. Several states with the highest cigarette smoking prevalence reported the lowest prevalence of interest in quitting, quit attempts, receipt of advice to quit, and use of counseling and/or medication, and the highest prevalence of e-cigarette, smokeless tobacco, and cigar use.

**Conclusion:**

Adults who smoke struggle with smoking cessation and could benefit from additional intervention.

**Summary**

**What is already known on this topic?**

Increasing quitting among people who use tobacco products is the quickest approach to reducing commercial tobacco-related disease and death.

**What is added by this report?**

In 2018–2019, past-year quit attempts ranged from 44.1% to 62.8% across states. Recent (past-year) successful smoking cessation ranged from 4.6% to 10.8%. Among adults who smoked and tried to quit, only 25.5% to 50.1% used evidence-based methods.

**What are the implications for public health practice?**

Adults who struggle with smoking cessation could benefit from additional intervention. Prevention opportunities exist at both individual (eg, community-cessation intervention programs) and population (eg, insurers covering cessation treatments; health systems integrating evidence-based cessation interventions into routine clinical care) levels.

**MEDSCAPE CME**

In support of improving patient care, this activity has been planned and implemented by Medscape, LLC and *Preventing Chronic Disease.* Medscape, LLC is jointly accredited with commendation by the Accreditation Council for Continuing Medical Education (ACCME), the Accreditation Council for Pharmacy Education (ACPE), and the American Nurses Credentialing Center (ANCC), to provide continuing education for the healthcare team.

Medscape, LLC designates this Journal-based CME activity for a maximum of 1.0 AMA PRA Category 1 Credit(s)™. Physicians should claim only the credit commensurate with the extent of their participation in the activity.

Successful completion of this CME activity, which includes participation in the evaluation component, enables the participant to earn up to 1.0 MOC points in the American Board of Internal Medicine’s (ABIM) Maintenance of Certification (MOC) program. Participants will earn MOC points equivalent to the amount of CME credits claimed for the activity. It is the CME activity provider’s responsibility to submit participant completion information to ACCME for the purpose of granting ABIM MOC credit.

**Release date: November 16, 2023; Expiration date: November 16, 2024**

**Learning Objectives**

Upon completion of this activity, participants will be able to:

1. Distinguish current patterns in tobacco use among US adults
2. Compare US regions regarding cigarette use and the desire to quit cigarette smoking
3. Analyze tobacco cessation beliefs and behaviors in the United States
4. Evaluate how US adults use evidence-based tools to help them quit smoking

**Credit Hours —** 1.0

Accreditation Statements

In support of improving patient care, this activity has been planned and implemented by Medscape, LLC and *Preventing Chronic Disease.* Medscape, LLC is jointly accredited with commendation by the Accreditation Council for Continuing Medical Education (ACCME), the Accreditation Council for Pharmacy Education (ACPE), and the American Nurses Credentialing Center (ANCC), to provide continuing education for the healthcare team.

Medscape, LLC designates this Journal-based CME activity for a maximum of 1.0 *AMA PRA Category 1 Credit(s)*™. Physicians should claim only the credit commensurate with the extent of their participation in the activity.

Successful completion of this CME activity, which includes participation in the evaluation component, enables the participant to earn up to 1.0 MOC points in the American Board of Internal Medicine's (ABIM) Maintenance of Certification (MOC) program. Participants will earn MOC points equivalent to the amount of CME credits claimed for the activity. It is the CME activity provider's responsibility to submit participant completion information to ACCME for the purpose of granting ABIM MOC credit.

EDITOR

Camille Martin, RD, Editor, Preventing Chronic Disease, Atlanta, Georgia

AUTHORS

​​​​Monica E. Cornelius, PhD, Office on Smoking and Health National Center for Chronic Disease Prevention and Health Promotion, Atlanta, Georgia

Teresa W. Wang, PhD, Office on Smoking and Health National Center for Chronic Disease Prevention and Health Promotion, Atlanta, Georgia

Ahmed Jamal, MBBS, Office on Smoking and Health National Center for Chronic Disease Prevention and Health Promotion, Atlanta, Georgia

Caitlin Loretan, MPH, Office on Smoking and Health National Center for Chronic Disease Prevention and Health Promotion, Atlanta, Georgia

Gordon Willis, PhD, Behavioral Research Program Division of Cancer Control and Population Sciences, National Cancer Institute, Rockville, Maryland

Bria Graham-Glover, MPH, Center for Tobacco Products, US Food and Drug Administration, Silver Spring, Maryland

Linda Neff, MPH, Office on Smoking and Health National Center for Chronic Disease Prevention and Health Promotion, Atlanta, Georgia

CME AUTHOR

Charles P. Vega, MD, Health Sciences Clinical Professor of Family Medicine, University of California, Irvine School of Medicine

Charles P. Vega, MD, has the following relevant financial relationships:

Consultant or advisor for: Boehringer Ingelheim Pharmaceuticals, Inc.; GlaxoSmithKline; Johnson & Johnson Pharmaceutical Research & Development, L.L.C.

## Introduction

Although commercial tobacco use has declined over the past few decades, it remains a significant cause of preventable disease and death in the US (1–4). Increasing the number of people who quit smoking cigarettes is the quickest approach to reducing tobacco-related disease, death, and health care costs (3,5). Smoking cessation represents a critical component of a comprehensive tobacco control program (3,5). State programs can increase smoking cessation through implementation of educational interventions and delivery of cessation services (3,6,7).

Variation in state tobacco prevention and control programs, demographic characteristics of adults who smoke cigarettes, and the changing landscape of available tobacco products may all affect cessation. A higher prevalence of current cigarette smoking has been reported among certain racial and ethnic groups (eg, non-Hispanic American Indian and Alaska Native populations), sexual-minority groups (eg, those who identify as LGBTQ), people with lower income, and people aged 25 to 54 years (1,2,8), all of which may differ by geographic area. Geographic differences in population groups with greater cessation needs, along with jurisdictional differences related to economics, health, and tobacco control laws, may all play a role in cessation success.

Indicators for smoking cessation can be used to gauge the extent to which individuals are quitting, how the extent to which individuals are quitting relates to state tobacco prevention and control measures, and how each differs between states.

To inform national and state efforts to increase smoking cessation and reduce commercial tobacco use, this study builds on work by Wang et al (9) and provides 2018–2019 national and state-specific prevalence estimates of current adult tobacco product use, in addition to providing updated prevalence estimates for the following cessation indicators: 1) interest in quitting smoking; 2) quit attempts within the past year; 3) recent successful smoking cessation (quitting for ≥6 months in the past year); 4) receipt of advice to quit from a medical doctor; and 5) use of cessation medication and/or counseling during the most recent past-year quit attempt.

## Methods

### Data source

Data came from the 2018–2019 Tobacco Use Supplement to the Current Population Survey (TUS-CPS). The CPS uses a multistage probability sample based on results of the US Census to interview a nationally representative sample of noninstitutionalized US civilians aged 18 years or older in all 50 states and the District of Columbia (DC) (10). The TUS is a cross-sectional household-based survey which is attached to the CPS every 3 to 4 years (11). The TUS-CPS is a key source of national and state-level data on tobacco-related use behaviors, attitudes, and policies (11). The 2018–2019 TUS-CPS was conducted by telephone or in person in 3 waves: July 2018, January 2019, and May 2019. Combined, 137,471 adults completed the interview as self-respondents, with an average self-response rate of 56%. Institutional review board approval was not required because TUS-CPS data are deidentified and publicly available. In this report, “tobacco” refers to commercial tobacco products and not to tobacco used for medicinal and spiritual purposes by some American Indian communities.

### Measures

Adults who currently smoke were defined as those aged 18 years or older who had smoked 100 or more cigarettes during their lifetime and reported smoking “every day” or “some days” at the time of interview. Adults who formerly smoked were defined as those who had smoked 100 or more cigarettes during their lifetime and reported smoking “not at all” at the time of interview.

Current use of cigars (cigars, cigarillos, little filtered cigars), regular pipes, water pipes or hookah, e-cigarettes, and smokeless tobacco products (chewing tobacco, snuff, dip, snus, or dissolvable products) was defined as use of each of these products “every day” or “some days” at the time of interview. Any combustible tobacco use was defined as current use of at least 1 combustible tobacco product (cigarettes; cigars, cigarillos, filtered little cigars; pipes, water pipes or hookah). Current use of any tobacco product was defined as current use of at least 1 tobacco product.

Interest in quitting was assessed among adults who currently smoke cigarettes and indicated their interest in quitting smoking by selecting a response on a 10-point scale, which ranged from 1 (not at all interested) to 10 (extremely interested). Those selecting a response from 2 to 10 were considered as being interested in quitting smoking (9).

Past-year quit attempts was assessed among adults who currently smoke cigarettes. Those who reported having stopped smoking for 1 or more days or reported having made a serious attempt to stop smoking (even <1 day) within the past year were classified as having made a quit attempt (12). Additionally, adults who formerly smoked and who quit within the past year were classified as having made a quit attempt (12).

Recent successful quitting was assessed among adults who currently smoke cigarettes and initiated smoking at least 2 years ago and adults who formerly smoked who reported quitting within the past year. Recent successful cessation was defined as remaining abstinent from smoking for 6 months or longer within the past year (12).

Past-year receipt of medical advice to quit was assessed among adults who currently smoke cigarettes who visited a medical doctor within the past year and adults who formerly smoked who visited a medical doctor within the year before they quit smoking. Those who reported receiving advice to quit smoking were considered as having received past-year advice to quit.

Adults who currently or formerly smoked cigarettes who answered yes to having used evidence-based medications (nicotine patch, gum, lozenge, nasal spray, inhaler, Chantix/varenicline, Zyban/bupropion/Wellbutrin) and/or counseling (telephone help line or quit line; one-on-one in-person counseling by a health professional; stop-smoking clinic; internet or web-based program or tool including smartphone apps and text messaging programs) during their last past-year quit attempt were considered as having used medications and/or counseling.

### Statistical analysis

Data were weighted to yield national and state-representative point prevalence estimates and 95% CIs for all 50 states and DC. Quartiles were mapped for each tobacco product use definition and each cessation indicator. Statistical analyses were performed using SAS-callable SUDAAN, version 11.0.1 (Research Triangle Institute). Unstable estimates, defined as a relative standard error (RSE) of more than 30% or an unweighted denominator of less than 50, were suppressed. The number of states and their US Census region designation falling within the lower and upper quartiles were identified (13).

## Results

### Tobacco product use

During 2018–2019, prevalence of current use of any tobacco product ranged from 10.2% (95% CI, 9.5%–10.8%) in California to 29.0% (95% CI, 25.0%–32.9%) in West Virginia, with a median of 16.5% (Table 1). Among the 13 states and federal district in the lowest quartile (≤14.0%), 5 were from the South (DC, Delaware, Florida, Texas, Virginia) and 5 were from the Northeast (Connecticut, Massachusetts, New Jersey, New York, Rhode Island). Among the 12 states in the highest quartile (≥20.1%), 6 states were from the South (Alabama, Arkansas, Kentucky, Mississippi, Oklahoma, West Virginia). The prevalence of any combustible tobacco product use ranged from 7.9% (95% CI, 6.0%–9.8%) in Utah to 22.9% (95% CI, 19.8%–26.0%) in West Virginia (Table 1).

**Table 1:** Prevalence of Current Use of Tobacco Products Among Adults Aged ≥18 Years, by State, Tobacco Use Supplement to the Current Population Survey, United States, 2018–2019^a^

State	Any tobacco^b^	Combustible tobacco products^c^	Cigarettes^d^	Cigars /cigarillos/ filtered little cigars^e^	Regular pipes^f^	Water pipes/ hookah^g^	E-cigarettes^h^	Smokeless tobacco^i^
	% (95% CI)							
**National**	**15.4 (15.1–15.6)**	**13.0 (12.8–13.3)**	**11.4 (11.2–11.6)**	**2.1 (2.0–2.2)**	**0.3 (0.2–0.3)**	**0.4 (0.4–0.5)**	**2.3 (2.2–2.4)**	**1.4 (1.4–1.5)**
Alabama	21.6 (19.6–23.6)	16.7 (15.1–18.4)	15.2 (13.6–16.8)	2.0 (1.3–2.7)	j	j	3.4 (2.6–4.3)	3.9 (3.2–4.7)
Alaska	18.4 (15.7–21.1)	16.5 (13.8–19.1)	14.3 (11.8–16.8)	2.4 (1.5–3.4)	j	j	2.2 (1.1–3.3)	2.2 (1.3–3.1)
Arizona	14.4 (12.7–16.2)	12.3 (10.8–13.8)	10.5 (9.1–11.9)	2.1 (1.5–2.8)	j	j	3.4 (2.3–4.5)	0.9 (0.5–1.2)
Arkansas	21.1 (17.7–24.6)	17.3 (14.4–20.2)	15.5 (12.7–18.4)	2.5 (1.7–3.3)	j	j	2.5 (1.6–3.4)	3.5 (2.5–4.5)
California	10.2 (9.5–10.8)	9.0 (8.4–9.7)	7.5 (7.0–8.1)	1.5 (1.2–1.7)	0.2 (0.1–0.3)	0.5 (0.3–0.7)	1.6 (1.3–2.0)	0.4 (0.3–0.5)
Colorado	14.8 (12.1–17.6)	11.5 (9.6–13.5)	9.9 (8.0–11.9)	1.7 (0.8–2.5)	j	j	3.2 (2.1–4.4)	j
Connecticut	12.9 (10.6–15.3)	11.5 (9.3–13.7)	9.4 (7.4–11.3)	2.7 (1.6–3.8)	j	j	2.0 (1.0–3.1)	j
Delaware	13.4 (11.1–15.8)	12.6 (10.2–14.9)	10.7 (8.6–12.8)	2.2 (1.1–3.2)	j	j	1.4 (0.8–2.1)	j
District of Columbia	14.0 (12.4–15.6)	13.2 (11.6–14.8)	9.9 (8.5–11.3)	2.6 (1.8–3.3)	j	2.0 (1.4–2.7)	1.3 (0.8–1.8)	0.4 (0.2–0.6)
Florida	13.9 (12.9–14.9)	12.4 (11.5–13.3)	10.6 (9.7–11.5)	2.1 (1.7–2.5)	0.4 (0.2–0.6)	0.3 (0.2–0.4)	1.7 (1.2–2.1)	0.8 (0.5–1.1)
Georgia	16.2 (14.6–17.9)	14.1 (12.5–15.6)	12.0 (10.5–13.5)	2.3 (1.7–2.9)	j	0.7 (0.4–1.1)	2.2 (1.5–3.0)	1.2 (0.6–1.7)
Hawaii	10.8 (8.9–12.7)	9.1 (7.4–10.8)	8.7 (7.0–10.3)	0.7 (0.3–1.1)	0 (0.0–0.0)	j	2.7 (1.6–3.9)	j
Idaho	16.3 (14.3–18.2)	12.4 (10.7–14.1)	10.8 (9.3–12.3)	1.7 (1.1–2.2)	j	j	2.8 (2.1–3.6)	3.2 (2.1–4.3)
Illinois	15.7 (14.1–17.3)	14.0 (12.5–15.6)	12.2 (10.7–13.7)	2.2 (1.6–2.8)	j	j	2.3 (1.8–2.8)	1.1 (0.6–1.5)
Indiana	20.0 (17.3–22.6)	16.8 (14.4–19.3)	14.7 (12.3–17.2)	2.9 (2.0–3.7)	j	j	3.0 (2.2–3.8)	2.1 (1.4–2.7)
Iowa	20.3 (17.3–23.4)	17.6 (14.6–20.7)	15.9 (13.2–18.6)	2.7 (1.7–3.6)	j	j	3.1 (2.1–4.1)	2.2 (1.5–2.9)
Kansas	18.6 (15.4–21.7)	14.9 (12.5–17.3)	12.3 (10.1–14.5)	3.3 (2.3–4.4)	j	j	2.7 (1.4–4.1)	2.3 (1.6–2.9)
Kentucky	24.8 (22.1–27.5)	20.4 (18.0–22.8)	18.3 (15.7–20.9)	2.4 (1.1–3.6)	j	j	3.5 (2.1–4.9)	4.3 (2.3–6.2)
Louisiana	18.9 (16.5–21.2)	17.0 (14.9–19.1)	14.9 (12.9–16.9)	2.8 (2.1–3.5)	j	j	1.7 (1.0–2.3)	1.5 (1.0–2.1)
Maine	20.2 (17.7–22.6)	17.9 (15.7–20.2)	15.9 (13.7–18.0)	2.6 (1.4–3.7)	j	j	3.3 (1.9–4.7)	0.9 (0.4–1.5)
Maryland	14.4 (12.4–16.5)	12.4 (10.4–14.3)	9.9 (8.2–11.5)	2.5 (1.6–3.3)	j	j	2.2 (1.5–3.0)	j
Massachusetts	11.2 (9.7–12.8)	10.0 (8.5–11.4)	8.4 (7.0–9.7)	1.7 (1.2–2.2)	j	j	1.8 (1.2–2.4)	j
Michigan	18.0 (16.1–20.0)	16.3 (14.4–18.2)	14.4 (12.6–16.2)	2.3 (1.7–2.9)	j	j	1.9 (1.4–2.5)	1.4 (0.9–1.9)
Minnesota	16.2 (13.5–19.0)	13.9 (11.1–16.8)	11.9 (9.6–14.1)	2.0 (1.1–2.9)	j	j	1.5 (0.9–2.1)	2.1 (1.4–2.8)
Mississippi	20.6 (19.0–22.2)	17.4 (15.9–18.9)	16.2 (15.0–17.5)	2.5 (1.7–3.2)	j	j	1.4 (0.9–2.0)	3.2 (2.4–3.9)
Missouri	19.1 (17.0–21.3)	16.0 (13.7–18.4)	13.5 (11.4–15.7)	2.5 (1.7–3.4)	j	j	2.7 (1.8–3.5)	2.2 (1.3–3.1)
Montana	19.9 (18.2–21.7)	15.8 (14.3–17.3)	14.5 (12.9–16.1)	2.0 (1.3–2.6)	0.2 (0.1–0.3)	j	2.4 (1.6–3.1)	3.8 (3.0–4.6)
Nebraska	18.8 (16.9–20.7)	15.7 (13.8–17.6)	13.8 (12.0–15.6)	2.6 (1.7–3.4)	j	j	3.2 (2.0–4.3)	1.7 (0.9–2.5)
Nevada	15.4 (13.5–17.3)	14.2 (12.4–16.1)	12.4 (10.6–14.1)	1.9 (1.1–2.7)	j	j	2.0 (1.1–2.9)	j
New Hampshire	17.4 (15.2–19.6)	15.5 (13.5–17.5)	12.4 (10.6–14.1)	3.0 (2.1–4.0)	j	j	2.0 (1.0–3.1)	0.7 (0.3–1.2)
New Jersey	11.7 (10.4–13.1)	10.4 (9.1–11.7)	7.8 (6.6–8.9)	2.5 (1.7–3.2)	j	0.8 (0.4–1.3)	2.1 (1.4–2.9)	j
New Mexico	15.5 (13.3–17.7)	12.6 (10.6–14.6)	11.2 (9.5–12.9)	1.6 (0.8–2.4)	j	j	2.5 (1.6–3.4)	1.7 (1.3–2.0)
New York	12.9 (12.0–13.9)	11.2 (10.3–12.2)	9.5 (8.7–10.2)	1.3 (1.0–1.6)	0.2 (0.1–0.2)	0.8 (0.5–1.2)	2.1 (1.7–2.5)	0.5 (0.3–0.8)
North Carolina	18.8 (16.8–20.7)	15.9 (14.3–17.6)	13.6 (12.0–15.1)	2.5 (1.8–3.3)	j	0.6 (0.3–0.9)	2.7 (2.1–3.3)	2.1 (1.6–2.7)
North Dakota	22.0 (19.4–24.6)	17.3 (15.1–19.5)	15.8 (13.7–17.9)	2.2 (1.5–2.9)	j	j	3.2 (2.2–4.1)	4.3 (2.8–5.7)
Ohio	21.6 (20.1–23.0)	18.7 (17.3–20.1)	16.4 (15.1–17.8)	3.1 (2.5–3.8)	j	j	2.8 (2.1–3.5)	2.1 (1.6–2.6)
Oklahoma	23.6 (21.6–25.6)	17.8 (15.8–19.7)	16.1 (14.2–18.1)	2.4 (1.7–3.2)	j	j	4.9 (3.8–6.1)	3.4 (2.4–4.4)
Oregon	16.0 (14.0–18.1)	13.2 (11.3–15.0)	11.2 (9.3–13.1)	1.9 (1.1–2.7)	j	j	3.2 (2.2–4.1)	1.9 (1.2–2.6)
Pennsylvania	18.5 (17.0–20.0)	16.2 (14.8–17.6)	13.7 (12.4–14.9)	2.8 (2.2–3.5)	0.4 (0.2–0.6)	j	2.4 (1.8–3.0)	1.8 (1.4–2.3)
Rhode Island	12.9 (10.6–15.3)	11.0 (9.1–12.9)	8.4 (6.8–10.0)	1.9 (1.0–2.8)	j	j	j	j
South Carolina	16.5 (14.5–18.5)	14.2 (12.2–16.2)	12.8 (10.9–14.8)	1.8 (1.2–2.4)	j	j	1.9 (1.3–2.6)	1.6 (1.1–2.2)
South Dakota	20.7 (18.6–22.9)	17.8 (15.5–20.0)	15.5 (13.2–17.7)	2.2 (1.3–3.1)	j	j	3.5 (2.3–4.7)	2.4 (1.6–3.1)
Tennessee	19.7 (18.2–21.2)	17.3 (15.9–18.7)	15.8 (14.4–17.1)	2.0 (1.4–2.6)	j	j	2.0 (1.2–2.8)	2.1 (1.6–2.6)
Texas	13.4 (12.6–14.2)	10.8 (10.1–11.6)	9.0 (8.3–9.8)	1.7 (1.3–2.1)	0.1 (0.1–0.2)	0.4 (0.2–0.6)	2.3 (1.9–2.7)	1.5 (1.2–1.8)
Utah	10.7 (8.6–12.8)	7.9 (6.0–9.8)	6.7 (5.0–8.3)	1.0 (0.4–1.5)	j	j	3.0 (2.1–3.8)	1.4 (0.8–2.0)
Vermont	14.2 (12.3–16.2)	12.5 (10.8–14.2)	10.8 (9.2–12.4)	1.8 (1.1–2.5)	j	j	1.9 (1.1–2.7)	1.0 (0.4–1.6)
Virginia	14.0 (12.0–16.0)	11.8 (9.9–13.6)	9.6 (7.8–11.4)	2.3 (1.7–3.0)	j	0.6 (0.3–0.9)	2.4 (1.7–3.0)	1.3 (0.8–1.7)
Washington	13.7 (11.8–15.7)	10.9 (9.5–12.2)	9.9 (8.7–11.2)	1.4 (0.8–1.9)	j	j	2.3 (1.5–3.1)	1.7 (0.9–2.5)
West Virginia	29.0 (25.0–32.9)	22.9 (19.8–26.0)	21.3 (18.5–24.1)	2.4 (1.5–3.3)	0.6 (0.3–0.9)	j	3.8 (2.9–4.7)	6.3 (4.8–7.8)
Wisconsin	17.8 (15.9–19.7)	14.9 (13.3–16.5)	13.2 (11.7–14.7)	1.9 (1.4–2.3)	j	j	2.6 (1.6–3.6)	1.8 (1.1–2.6)
Wyoming	22.6 (19.6–25.5)	16.2 (14.2–18.3)	14.2 (12.2–16.2)	2.2 (1.5–2.9)	j	j	3.7 (2.6–4.7)	5.2 (3.0–7.3)

a In this article, “tobacco” refers to commercial tobacco products and not to tobacco used for medicinal and spiritual purposes by some American Indian communities.

b Any tobacco use was defined as use either “every day” or “some days” of at least 1 tobacco product. For cigarettes, users were defined as adults who reported use either “every day” or “some days” and had smoked ≥100 cigarettes during their lifetime.

c Any combustible tobacco use was defined as use either “every day” or “some days” of at least 1 combustible tobacco product: cigarettes; cigars, cigarillos, or filtered little cigars; and pipes, water pipes, or hookah. For cigarettes, users were defined as adults who reported use either “every day” or “some days” and had smoked ≥100 times during their lifetime.

d Adults who currently smoke cigarettes were defined as adults who reported smoking ≥100 cigarettes during their lifetime and now smoked cigarettes “every day” or “some days.”

e Adults who currently smoke cigars were defined as adults who currently reported smoking cigars, cigarillos, or little filtered cigars “every day” or “some days.”

f Adults who currently smoke pipes were defined as adults who reported currently smoking tobacco in a regular pipe “every day” or “some days.”

g Adults who currently smoke waterpipes or hookahs were defined as adults who reported currently smoking tobacco in a waterpipe or hookah “every day” or “some days.”

h Adults who currently use e-cigarettes were defined as adults who reported using e-cigarettes at least once during their lifetime and now use e-cigarettes “every day” or “some days.”

i Current smokeless tobacco product users were defined as adults who reported using chewing tobacco, snuff, dip, snus, or dissolvable tobacco at least once during their lifetime and now use at least 1 of these products “every day” or “some days.”

j Unweighted denominator <50 or relative standard error >30%.

Prevalence of current cigarette smoking ranged from 6.7% (95% CI, 5.0%–8.3%) in Utah to 21.3% (95% CI, 18.5%–24.1%) in West Virginia, with a median of 12.4% (Table 1). Among the 14 states in the lowest quartile (≤9.9%), 5 were in the West (California, Colorado, Hawaii, Utah, Washington) and 5 were from the Northeast (Connecticut, Massachusetts, New Jersey, New York, Rhode Island). Among the 13 states in the highest quartile (≥14.9%), 8 were from the South (Alabama, Arkansas, Kentucky, Louisiana, Mississippi, Oklahoma, Tennessee, West Virginia).

The prevalence of e-cigarette use ranged from 1.3% (95% CI, 0.8%–1.8%) in DC to 4.9% (95% CI, 3.8%–6.1%) in Oklahoma, with a median of 2.4% (Table 1). Among the 14 states and federal district in the lowest quartile (≤2.0%), 7 were from the South (DC, Delaware, Florida, Louisiana, Mississippi, South Carolina, Tennessee). Among the 12 states in the highest quartile (≥3.2%), 4 were from the South (Alabama, Kentucky, Oklahoma, West Virginia) and 4 were from the West (Arizona, Colorado, Oregon, Wyoming).

The prevalence of cigar smoking (including cigarillos and filtered little cigars) ranged from 0.7% (95% CI, 0.3%–1.1%) in Hawaii to 3.3% (95% CI, 2.3%–4.4%) in Kansas, with a median of 2.2% (Table 1). The prevalence of smokeless tobacco product use ranged from 0.4% (95% CI, 0.3%–0.5%) in California and 0.4% (95% CI, 0.2%–0.6%) in DC to 6.3% (95% CI, 4.8%–7.8%) in West Virginia, with a median of 1.9% (Table 1). The prevalence of pipe use ranged from 0% in Hawaii to 0.6% (95% CI, 0.3%–0.9%) in West Virginia, with a median of 0.2% (Table 1). The prevalence of water pipe or hookah use ranged from 0.3% (95% CI, 0.2%–0.4%) in Florida to 2.0% (95% CI, 1.4%–2.7%) in DC, with a median of 0.6% (Table 1).

### Cessation indicators

The prevalence of adults who currently smoke cigarettes reporting they were interested in quitting ranged from 68.2% (95% CI, 63.5%–72.9%) in Alabama to 87.5% (95% CI, 81.0%–94.0%) in Connecticut, with a median of 76.5% (Table 2). Among the 13 states in the lowest quartile (≤73.7%), 7 were in the South (Alabama, Arkansas, Florida, Kentucky, Mississippi, Oklahoma, West Virginia); among the 12 states in the highest quartile (≥79.3%), 5 were in the Northeast (Connecticut, New Hampshire, New Jersey, Pennsylvania, Rhode Island) (Figure, Map A).

**Table 2:** State-Specific Prevalence of Smoking Cessation and Cessation Treatment Indicators, Tobacco Use Supplement to the Current Population Survey, United States, 2018–2019^a^^,^ b

State	Interested in quitting^c^	Past-year quit attempts^d^	Recent smoking cessation^e^	Receipt of advice to quit^f^	Use of cessation counseling and/or medications to quit^g^	Use of counseling to quit^h^	Use of cessation medications to quit^i^
	% (95% CI)						
**National**	**76.6 (75.8–77.4)**	**51.9 (51.1–52.8)**	**7.4 (7.0–7.9)**	**71.8 (70.8–72.9)**	**34.3 (33.1–35.6)**	**10.2 (9.5–10.9)**	**31.0 (29.8–32.2)**
Alabama	68.2 (63.5–72.9)	46.1 (39.8–52.4)	5.0 (3.0–7.0)	71.1 (63.3–78.9)	27.7 (23.3–32.1)	6.9 (3.7–10.1)	25.7 (21.4–30.0)
Alaska	72.2 (64.1–80.3)	53.0 (44.8–61.2)	7.8 (3.6–12.0)	72.8 (64.7–80.9)	48.1 (38.1–58.1)	24.0 (12.8–35.2)	44.3 (35.1–53.5)
Arizona	78.4 (72.6–84.2)	53.8 (46.9–60.7)	7.7 (4.7–10.7)	63.5 (54.9–72.1)	31.3 (23.8–38.8)	12.6 (7.1–18.1)	27.7 (20.5–34.9)
Arkansas	72.7 (68.3–77.1)	45.1 (40.0–50.2)	6.8 (3.8–9.8)	65.2 (59.1–71.3)	44.6 (36.5–52.7)	8.8 (4.8–12.8)	41.5 (33.1–49.9)
California	80.0 (76.7–83.3)	54.4 (50.6–58.2)	9.0 (7.0–11.0)	69.0 (64.7–73.3)	30.1 (25.3–34.9)	13.0 (9.6–16.4)	26.7 (22.1–31.3)
Colorado	83.6 (75.7–91.5)	54.2 (45.0–63.4)	j	63.3 (51.9–74.7)	29.8 (18.2–41.4)	j	27.8 (16.1–39.5)
Connecticut	87.5 (81.0–94.0)	52.0 (41.9–62.1)	j	83.5 (75.4–91.6)	44.8 (32.0–57.6)	j	38.9 (26.1–51.7)
Delaware	78.4 (69.6–87.2)	57.2 (49.0–65.4)	7.9 (3.9–11.9)	84.4 (77.4–91.4)	30.8 (20.3–41.3)	j	27.7 (18.0–37.4)
District of Columbia	77.3 (70.1–84.5)	54.8 (47.8–61.8)	8.5 (4.9–12.1)	76.5 (69.8–83.2)	31.7 (24.0–39.4)	11.3 (6.4–16.2)	27.9 (20.4–35.4)
Florida	73.1 (68.8–77.4)	49.1 (44.9–53.3)	6.7 (4.6–8.8)	72.7 (67.3–78.1)	32.5 (27.0–38.0)	13.7 (9.7–17.7)	28.5 (23.4–33.6)
Georgia	79.2 (74.7–83.7)	44.9 (39.2–50.6)	5.9 (2.7–9.1)	76.3 (69.5–83.1)	27.4 (19.9–34.9)	11.1 (6.6–15.6)	24.8 (17.4–32.2)
Hawaii	78.1 (71.7–84.5)	55.7 (46.0–65.4)	j	70.4 (59.1–81.7)	29.1 (17.3–40.9)	j	24.9 (14.3–35.5)
Idaho	76.0 (71.1–80.9)	55.4 (49.3–61.5)	9.8 (6.2–13.4)	68.4 (60.8–76.0)	35.0 (25.8–44.2)	8.8 (4.1–13.5)	33.4 (24.4–42.4)
Illinois	77.2 (72.9–81.5)	52.5 (47.9–57.1)	8.3 (5.4–11.2)	73.3 (67.2–79.4)	37.1 (29.9–44.3)	12.0 (8.1–15.9)	31.9 (24.5–39.3)
Indiana	73.9 (69.8–78.0)	54.8 (49.1–60.5)	9.9 (6.8–13.0)	75.1 (67.7–82.5)	36.9 (28.8–45.0)	11.0 (5.5–16.5)	29.8 (22.7–36.9)
Iowa	69.5 (61.8–77.2)	49.9 (44.8–55.0)	j	75.0 (66.6–83.4)	38.1 (26.1–50.1)	j	35.1 (24.1–46.1)
Kansas	72.5 (65.4–79.6)	52.0 (42.5–61.5)	10.3 (6.2–14.4)	65.5 (55.9–75.1)	27.4 (17.6–37.2)	j	27.2 (17.5–36.9)
Kentucky	68.8 (62.0–75.6)	46.3 (41.7–50.9)	4.8 (2.5–7.1)	69.9 (64.8–75.0)	28.8 (20.4–37.2)	7.2 (3.8–10.6)	27.5 (19.3–35.7)
Louisiana	76.0 (71.7–80.3)	55.7 (50.3–61.1)	7.5 (5.6–9.4)	67.0 (61.1–72.9)	32.0 (26.6–37.4)	10.7 (7.2–14.2)	29.2 (23.6–34.8)
Maine	75.4 (69.3–81.5)	54.3 (48.1–60.5)	8.2 (4.5–11.9)	71.5 (62.2–80.8)	45.9 (36.7–55.1)	12.9 (6.8–19.0)	43.1 (34.0–52.2)
Maryland	84.7 (79.0–90.4)	47.8 (38.6–57.0)	j	74.3 (64.8–83.8)	38.6 (27.4–49.8)	j	35.8 (25.2–46.4)
Massachusetts	77.9 (72.3–83.5)	56.2 (49.5–62.9)	9.8 (5.5–14.1)	77.3 (70.5–84.1)	50.1 (41.0–59.2)	7.6 (3.4–11.8)	47.5 (38.7–56.3)
Michigan	74.0 (67.4–80.6)	52.6 (47.3–57.9)	6.9 (4.1–9.7)	76.3 (70.7–81.9)	36.4 (29.4–43.4)	13.0 (8.3–17.7)	30.1 (24.1–36.1)
Minnesota	77.5 (72.3–82.7)	51.2 (44.7–57.7)	7.2 (4.0–10.4)	74.1 (67.8–80.4)	39.1 (29.8–48.4)	j	38.1 (28.5–47.7)
Mississippi	72.1 (67.1–77.1)	48.8 (41.0–56.6)	6.8 (3.9–9.7)	68.4 (61.2–75.6)	27.8 (22.9–32.7)	6.3 (2.6–10.0)	25.7 (21.1–30.3)
Missouri	73.8 (68.2–79.4)	49.1 (42.9–55.3)	8.1 (3.9–12.3)	64.8 (58.4–71.2)	34.4 (26.0–42.8)	j	31.7 (23.6–39.8)
Montana	75.8 (70.9–80.7)	50.5 (44.1–56.9)	5.4 (3.0–7.8)	73.3 (67.8–78.8)	39.8 (33.1–46.5)	11.7 (7.7–15.7)	37.9 (31.6–44.2)
Nebraska	82.5 (77.5–87.5)	53.7 (45.2–62.2)	6.4 (3.3–9.5)	66.9 (60.3–73.5)	28.0 (21.1–34.9)	9.1 (4.6–13.6)	23.2 (17.0–29.4)
Nevada	85.2 (80.7–89.7)	46.7 (38.6–54.8)	7.4 (3.9–10.9)	66.1 (57.2–75.0)	25.5 (17.3–33.7)	j	23.9 (16.1–31.7)
New Hampshire	87.4 (82.4–92.4)	55.1 (47.5–62.7)	5.1 (2.6–7.6)	81.8 (75.4–88.2)	41.4 (33.3–49.5)	12.0 (6.5–17.5)	38.0 (29.8–46.2)
New Jersey	80.4 (74.6–86.2)	57.3 (51.0–63.6)	6.3 (3.0–9.6)	79.0 (72.0–86.0)	32.3 (23.4–41.2)	j	29.8 (20.9–38.7)
New Mexico	75.5 (70.6–80.4)	53.1 (45.3–60.9)	8.1 (5.4–10.8)	64.5 (56.4–72.6)	34.3 (29.0–39.6)	14.5 (9.8–19.2)	30.7 (24.7–36.7)
New York	78.0 (73.7–82.3)	55.5 (51.6–59.4)	8.4 (5.8–11.0)	75.6 (71.2–80.0)	35.0 (28.7–41.3)	10.1 (6.4–13.8)	32.4 (26.0–38.8)
North Carolina	79.2 (75.2–83.2)	53.7 (48.3–59.1)	7.1 (4.5–9.7)	75.9 (69.3–82.5)	31.2 (24.7–37.7)	6.2 (3.1–9.3)	29.1 (23.3–34.9)
North Dakota	73.2 (67.8–78.6)	53.8 (47.1–60.5)	8.0 (4.2–11.8)	68.3 (62.1–74.5)	28.2 (20.6–35.8)	12.4 (6.1–18.7)	25.5 (17.6–33.4)
Ohio	72.0 (68.0–76.0)	51.7 (47.7–55.7)	6.6 (4.7–8.5)	68.2 (62.5–73.9)	34.2 (28.4–40.0)	6.1 (3.5–8.7)	32.4 (26.8–38.0)
Oklahoma	73.6 (66.9–80.3)	54.9 (49.2–60.6)	7.7 (5.3–10.1)	68.5 (61.0–76.0)	35.7 (29.7–41.7)	13.3 (7.4–19.2)	29.8 (24.7–34.9)
Oregon	83.7 (78.9–88.5)	58.0 (51.6–64.4)	8.4 (5.1–11.7)	71.6 (62.2–81.0)	39.5 (30.5–48.5)	9.8 (4.6–15.0)	37.0 (28.6–45.4)
Pennsylvania	80.3 (76.2–84.4)	56.2 (51.7–60.7)	6.5 (4.2–8.8)	68.8 (64.1–73.5)	39.5 (33.6–45.4)	10.6 (6.6–14.6)	33.0 (27.1–38.9)
Rhode Island	87.3 (81.1–93.5)	62.8 (53.2–72.4)	j	86.9 (79.6–94.2)	26.0 (15.8–36.2)	j	25.0 (14.7–35.3)
South Carolina	74.7 (68.4–81.0)	47.5 (40.4–54.6)	5.5 (2.4–8.6)	73.5 (67.2–79.8)	46.1 (35.5–56.7)	13.5 (6.2–20.8)	42.8 (32.3–53.3)
South Dakota	78.9 (71.1–86.7)	58.0 (52.6–63.4)	10.8 (6.9–14.7)	71.1 (64.6–77.6)	29.3 (17.6–41.0)	14.9 (7.2–22.6)	27.2 (16.3–38.1)
Tennessee	74.0 (69.8–78.2)	44.1 (39.7–48.5)	7.1 (4.7–9.5)	77.7 (72.2–83.2)	28.0 (21.7–34.3)	5.8 (2.8–8.8)	25.5 (19.4–31.6)
Texas	76.1 (72.5–79.7)	50.8 (46.7–54.9)	9.0 (6.7–11.3)	66.0 (61.4–70.6)	31.2 (26.0–36.4)	9.0 (6.0–12.0)	28.1 (23.2–33.0)
Utah	77.5 (69.4–85.6)	62.7 (54.9–70.5)	j	68.3 (57.8–78.8)	28.4 (17.5–39.3)	j	28.4 (17.5–39.3)
Vermont	78.8 (72.6–85.0)	53.1 (45.3–60.9)	j	71.1 (64.4–77.8)	49.4 (39.7–59.1)	16.8 (8.6–25.0)	47.5 (38.1–56.9)
Virginia	75.1 (68.3–81.9)	55.1 (49.1–61.1)	7.9 (3.9–11.9)	73.0 (64.7–81.3)	37.8 (28.6–47.0)	10.0 (5.6–14.4)	34.9 (25.0–44.8)
Washington	76.5 (71.1–81.9)	49.6 (43.4–55.8)	7.8 (4.4–11.2)	71.5 (65.2–77.8)	42.7 (34.5–50.9)	13.2 (7.3–19.1)	39.2 (31.1–47.3)
West Virginia	70.7 (65.8–75.6)	46.4 (39.9–52.9)	4.6 (2.9–6.3)	75.7 (70.4–81.0)	33.3 (27.6–39.0)	14.1 (10.7–17.5)	30.9 (25.3–36.5)
Wisconsin	79.4 (74.7–84.1)	50.3 (44.4–56.2)	4.6 (2.2–7.0)	79.5 (73.6–85.4)	35.7 (27.2–44.2)	8.5 (3.7–13.3)	35.0 (26.9–43.1)
Wyoming	73.6 (67.0–80.2)	53.1 (48.3–57.9)	10.0 (7.3–12.7)	64.5 (58.6–70.4)	37.2 (29.8–44.6)	12.0 (7.9–16.1)	33.1 (25.6–40.6)

a Adults who currently smoke cigarettes were defined as adults who reported smoking ≥100 cigarettes during their lifetime and now smoked cigarettes “every day” or “some days.”

b Adults who formerly smoked cigarettes were defined as adults who had smoked ≥100 cigarettes during their lifetime and reported smoking “not at all” at the time of interview.

c Adults who currently smoke cigarettes and who indicated their interest in quitting smoking by selecting a response from 2 to 10 on a 10-point scale, which ranged from 1 (not at all interested) to 10 (extremely interested).

d Adults who currently smoke cigarettes and who made a quit attempt in the past year who reported having stopped smoking for ≥1 days or reported having made a serious attempt to stop smoking (even <1 day) within the past year, and adults who formerly smoked who quit within the past year were classified as having made a quit attempt.

e Recent successful cessation was defined as adults who currently smoke and adults who formerly smoked who remained quit for ≥6 months within the past year. Recent successful cessation was assessed among adults who currently smoke and who initiated smoking at least 2 years ago, and adults who formerly smoked who reported quitting within the past year.

f Among adults who currently smoke who visited a medical doctor within the past year and adults who formerly smoked who visited a medical doctor within the year before they quit smoking, those who reported receiving advice to quit were considered as having received past-year advice to quit.

g Adults who currently smoke and adults who formerly smoked who answered yes to having used evidence-based medications (ie, nicotine patch, gum, lozenge, nasal spray, inhaler, Chantix/varenicline, Zyban/bupropion/Wellbutrin) and/or counseling (telephone help line or quit line; one-on-one in-person counseling by a health professional; stop-smoking clinic; internet or web-based program or tool, including smartphone apps and text messaging programs) during their last past-year quit attempt were classified as having used medications and/or counseling. We are not able to distinguish those who selected each item among those who selected “internet or web-based program or tool including smartphone apps and text messaging programs” and acknowledge the limitations in the definition for evidence-based counseling since the evidence is currently inadequate to infer that smartphone apps for smoking cessation are independently effective in increasing smoking cessation. See page 33 of the Surgeon General’s Report on Cessation (3).

h Adults who currently smoke and adults who formerly smoked who answered “yes” to having received counseling (telephone help line or quitline; one-on-one in-person counseling by a health professional; stop-smoking clinic; internet or web-based program or tool including smartphone apps and text messaging programs) during their last past-year quit attempt were considered as having used counseling to quit.

i Adults who currently smoke and adults who formerly smoked who answered yes to having used evidence-based medications (ie, nicotine patch, gum, lozenge, nasal spray, inhaler, Chantix/varenicline, Zyban/bupropion/Wellbutrin) during their last past-year quit attempt were considered as having used medications.

j Unweighted denominator <50 or relative standard error >30%.

**Figure:**
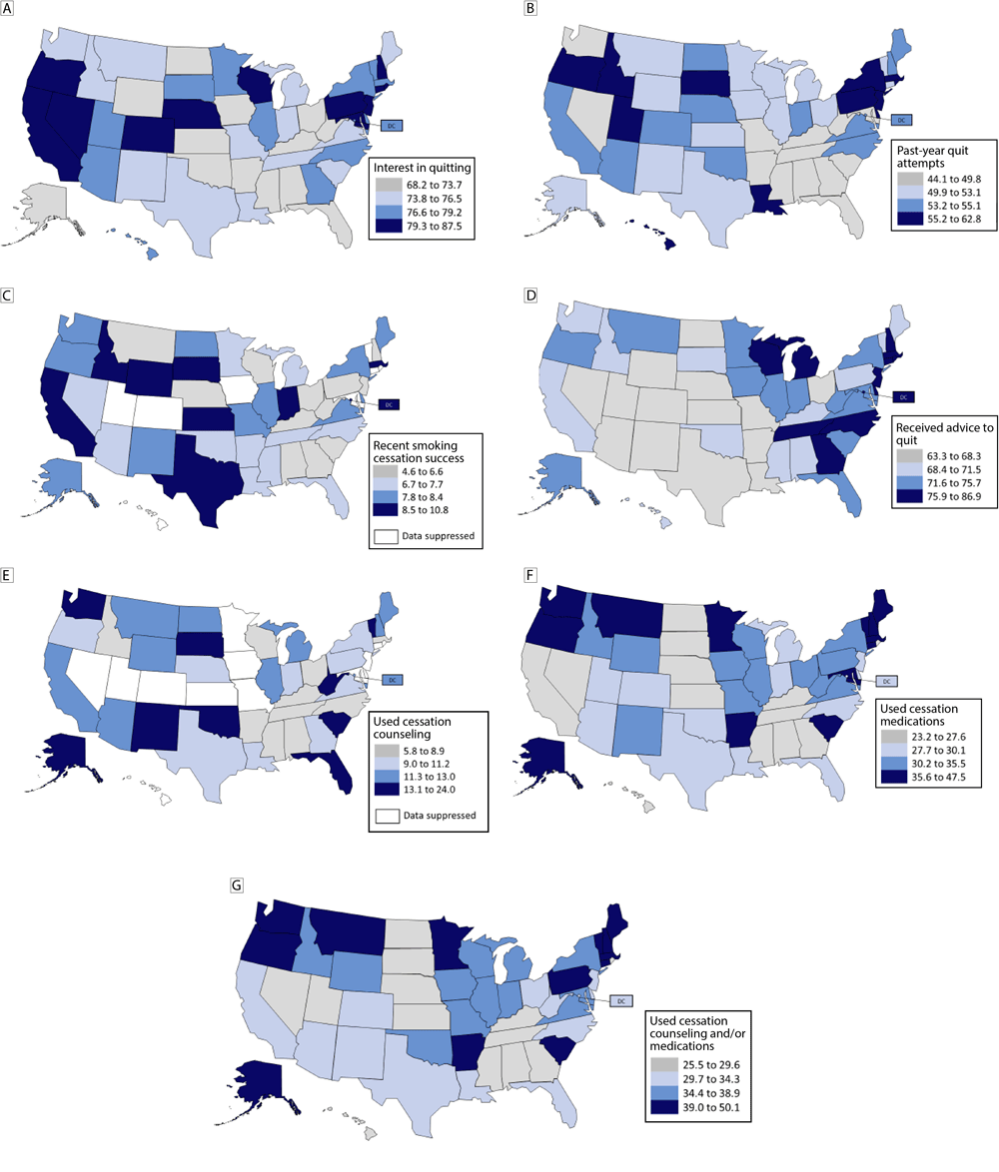
State-level prevalence of smoking cessation and cessation treatment indicators among adults aged ≥18 years who reported currently or formerly smoking cigarettes, by quartile, Tobacco Use Supplement to the Current Population Survey, United States, 2018–2019. All categories are defined in the Methods section. [A text description of this figure is available.]

The prevalence of adults who currently smoke or formerly smoked and reported past-year quit attempts ranged from 44.1% (95% CI, 39.7%–48.5%) in Tennessee to 62.8% (95% CI, 53.2%–72.4%) in Rhode Island, with a median of 53.1% (Table 2). Among the 13 states in the lowest quartile (≤49.8%), 10 were in the South (Alabama, Arkansas, Florida, Georgia, Kentucky, Maryland, Mississippi, South Carolina, Tennessee, West Virginia); among the 12 states in the highest quartile (≥55.2%), 5 were in the Northeast (Massachusetts, New Jersey, New York, Pennsylvania, Rhode Island) (Figure, Map B).

The prevalence of adults who smoked and recently successfully quit ranged from 4.6% (95% CI, 2.9%–6.3%) in West Virginia and 4.6% (95% CI, 2.2%–7.0%) in Wisconsin to 10.8% (95% CI, 6.9%–14.7%) in South Dakota, with a median value of 7.7% (Table 2). Of the 12 states in the lowest quartile (≤6.6%), 5 were in the South (Alabama, Georgia, Kentucky, South Carolina, West Virginia); of the 9 states in the highest quartile (≥8.5%), 3 were in the West (California, Idaho, Wyoming) and 3 were in the Midwest (Indiana, Kansas, South Dakota) (Figure, Map C).

The prevalence of adults who smoked and received advice to quit from a medical doctor ranged from 63.3% (95% CI, 51.9%–74.7%) in Colorado to 86.9% (95% CI, 79.6%–94.2%) in Rhode Island, with a median of 71.5% (Table 2). Among the 14 states in the lowest quartile (≤68.3%), 6 were in the West (Arizona, Colorado, Nevada, New Mexico, Utah, Wyoming); among the 11 states and federal district in the highest quartile (≥75.8%), 5 were in the South (DC, Delaware, Georgia, North Carolina, Tennessee) and 5 were in the Northeast (Connecticut, Massachusetts, New Hampshire, New Jersey, Rhode Island) (Figure, Map D).

The prevalence of adults who smoke who reported using cessation counseling during their last quit attempt ranged from 5.8% (95% CI, 2.8%–8.8%) in Tennessee to 24.0% (95% CI, 12.8%–35.2%) in Alaska, with a median of 11.2% (Table 2). Among the 10 states in the lowest quartile (≤8.9%), 6 were in the South (Alabama, Arkansas, Kentucky, Mississippi, North Carolina, Tennessee) and among the 9 states in the highest quartile (≥13.1%), 4 were in the South (Florida, Oklahoma, South Carolina, West Virginia) (Figure, Map E).

The prevalence of adults who smoke who reported using cessation medications during their last quit attempt ranged from 23.2% (95% CI, 17.0%–29.4%) in Nebraska to 47.5% (95% CI, 38.1%–56.9%) in Vermont and 47.5% (95% CI, 38.7%–56.3%) in Massachusetts, with a median of 30.1% (Table 2). Of the 13 states in the lowest quartile (≤27.6%), 5 were in the South (Alabama, Georgia, Kentucky, Mississippi, Tennessee), and of 13 states in the highest quartile (≥35.6%), 5 were in the Northeast (Connecticut, Maine, Massachusetts, New Hampshire, Vermont) (Figure, Map F).

The prevalence of adults who smoke and reported using counseling and/or medications during their last quit attempt ranged from 25.5% (95% CI, 17.3%–33.7%) in Nevada to 50.1% (95% CI, 41.0%–59.2%) in Massachusetts, with a median of 34.3% (Table 2). Of 13 states in the lowest quartile (≤29.6%), 5 were in the South (Alabama, Georgia, Kentucky, Mississippi, Tennessee), and of 13 states in the highest quartile (≥39.0%), 6 were in the Northeast (Connecticut, Maine, Massachusetts, New Hampshire, Pennsylvania, Vermont) (Figure, Map G).

Among the 13 states in the highest quartile for cigarette smoking, 7 were also in the highest quartile for e-cigarette use (Alabama, Kentucky, Maine, North Dakota, Oklahoma, South Dakota, West Virginia); 7 were in the highest quartile for smokeless use (Alabama, Arkansas, Kentucky, Mississippi, North Dakota, Oklahoma, West Virginia), and 4 were in the highest quartile for cigar use (Iowa, Louisiana, Maine, Ohio). Six states (Alabama, Kentucky, Maine, North Dakota, Oklahoma, West Virginia) were in the highest quartile for 3 or more tobacco products.

Among the 13 states in the highest quartile for cigarette smoking, 9 were in the lowest quartile for interest in quitting (Alabama, Arkansas, Iowa, Kentucky, Mississippi, North Dakota, Ohio, Oklahoma, West Virginia); 6 were in the lowest quartile for quit attempts (Alabama, Arkansas, Kentucky, Mississippi, Tennessee, West Virginia); 4 were in the lowest quartile for recent quit success (Alabama, Kentucky, Ohio, West Virginia); 4 were among the lowest quartile for receipt of advice to quit (Arkansas, Louisiana, North Dakota, Ohio); and 6 were among the lowest quartile for reporting use of counseling and/or medications to quit (Alabama, Kentucky, Mississippi, North Dakota, South Dakota, Tennessee).

## Discussion

During 2018–2019, variation existed in the prevalence of current commercial tobacco product use and cigarette smoking cessation behaviors among adults across US states. In every state, at least 10% of the adult population used at least 1 tobacco product, and combustible products (primarily cigarettes) were the most prevalent product used. More than two-thirds of adults who currently smoke cigarettes in all states and DC expressed at least some interest in quitting smoking. Similar to results from analysis of 2014–2015 TUS-CPS data (9), 3 in 10 people who smoked made no past-year quit attempts, 9 in 10 people who smoked did not successfully quit, and at least 1 in 10 people who smoked did not receive advice to quit from a medical doctor during a health care visit within the past year (9). Use of medications and/or counseling during the most recent past-year quit attempt was reported by one-quarter to one-half of all people who smoke. Except for receipt of advice to quit from a medical doctor, one-third to one-half of states in the lowest quartiles for all other cessation indicators assessed in this study were in the South. Several states with the highest prevalence of cigarette smoking also had a high prevalence of e-cigarette, smokeless tobacco, and cigar use and the lowest prevalence for interest in quitting, quit attempts, receiving advice to quit, and receipt of counseling and/or medications to quit.

Results suggest that adults who live in states with a higher prevalence of commercial tobacco use report less quit interest, cessation-focused behaviors, and more missed opportunities for cessation intervention from medical doctors. This variation between states may reflect jurisdictional differences in tobacco product use, demographic composition, tobacco prevention and control strategies, and access to cessation supports (5). For example, 9 of the 13 states with the highest prevalence of cigarette smoking have been noted as having weaker tobacco control policies and programs and disproportionately higher numbers of populations with more significant health care and financial needs (14). Social norms that influence perceptions toward tobacco use may also differ both between and within US Census regions (15). Taken together, these factors suggest that the variation in the prevalence of cessation indicators across states may be related to differences in implementation of strategies or access to cessation support and resources across states.

Estimates for interest in quitting smoking, quit attempts, recent successful cessation, receipt of advice to quit from a medical doctor, and use of evidence-based methods for quitting were consistent with previous estimates from national and state surveys, further highlighting difficulties in quitting smoking as a public health concern (3,8,16–18). Although most people who smoke cigarettes have made recent quit attempts, few have successfully quit. Only one-quarter to one-half of those who attempted to quit smoking within the past year used evidence-based cessation methods on their last quit attempt, with a higher prevalence reporting medication use than counseling.

The discrepancy between trying to quit and subsequently succeeding indicates opportunities for intervention. Use of evidence-based cessation methods represents an important area for improving quit rates, given that use of both counseling and medication together further increases cessation success (3,19). Use of quitlines also provides an affordable option for obtaining both behavioral counseling for nicotine addiction and access to cessation medications (3,5).

Behavioral interventions and cessation medications are key strategies for helping people who smoke to quit. However, population-level strategies can serve as an important complement to individual-level strategies (3). The 2020 US Surgeon General’s Report on smoking cessation states that increasing smoking cessation will require several strategies including 1) increasing the appeal, reach, and use of existing evidence-based cessation interventions; 2) further increasing the effectiveness of those interventions; and 3) developing additional cessation interventions that have greater reach and effectiveness than existing interventions or that appeal to and are used by different populations of people who smoke (3).

The path to successful smoking cessation is dynamic, challenging, and influenced by multiple behavioral, social, and biological factors (3). Tailoring evidence-based comprehensive tobacco control strategies to the needs of populations within jurisdictions can help with increasing cessation success and minimize geographical disparities (20). For example, states with higher proportions of populations with high tobacco use prevalence could tailor strategies to these groups (20). Using population-level interventions that affect social norms related to tobacco use could help states increase cessation by creating a social environment that denormalizes tobacco product use (eg, mass-reach intensive media campaigns to directly influence social norms; enactment of smoke-free laws; price increases) and provide greater opportunities for cessation (eg, offering free nicotine replacement therapy) (5).

### Limitations and strengths

The findings in this study are subject to at least 3 limitations. First, data were self-reported and are subject to recall and social desirability bias. Related to this, smoking and smoking cessation were not biochemically validated by serum cotinine measures. However, studies have shown that serum cotinine correlates well with self-reported smoking status (21). Second, this study does not fully address how other demographic, environmental, policy, and social factors may have influenced the geographic variation observed. Finally, this study focuses on state-specific prevalence and cannot account for within-state differences.

This study also has strengths. First, it provides state-level estimates of several key cessation indicators and use of evidence-based cessation methods. Although previous reports have provided only national-level estimates, reported only on some of the indicators at the state level, or used older information, this study provides 2018–2019 data using a broad set of cessation indicators (9,16,22). Additionally, this study provides an overall summary of results of combined state-specific tobacco product use and cessation indicator prevalence and compares this information by region. We have not found similar combined information in the published literature at the time of this writing. Second, this study provides state-specific estimates on 4 cessation behavioral indicators that align with Healthy People 2030 Tobacco Use Objectives 11 through 14, which specify national objectives related to quit attempts, receipt of advice to quit smoking from a medical doctor, use of smoking cessation counseling and/or medications, and recent cessation success, respectively (12). Finally, results of this report can be used to assist in monitoring state progress toward smoking cessation goals.

### Conclusion

Smoking cessation is a core component of comprehensive commercial tobacco control programs. Up-to-date state-level information on the prevalence of adult smoking cessation behaviors and on variations in these indicators across states are important for informing national and state efforts to increase delivery and use of proven cessation interventions. Prevention opportunities exist at both individual (eg, community cessation intervention programs) and population (eg, insurers covering cessation treatments; health systems integrating evidence-based cessation interventions into routine clinical care) levels (3–5). Use of state-level information on smoking prevalence and cessation behaviors can assist in identifying jurisdictions with the greatest need for cessation support. Identifying jurisdictions with the greatest cessation needs is a necessary step toward improving implementation of evidence-based strategies to increase cessation success and reduce overall commercial tobacco use.

## Post-Test Information

To obtain credit, you should first read the journal article. After reading the article, you should be able to answer the following, related, multiple-choice questions. To complete the questions (with a minimum 75% passing score) and earn continuing medical education (CME) credit, please go to http://www.medscape.org/journal/pcd. Credit cannot be obtained for tests completed on paper, although you may use the worksheet below to keep a record of your answers.

You must be a registered user on http://www.medscape.org. If you are not registered on http://www.medscape.org, please click on the “Register” link on the right hand side of the website.

Only one answer is correct for each question. Once you successfully answer all post-test questions, you will be able to view and/or print your certificate. For questions regarding this activity, contact the accredited provider, CME@medscape.net. For technical assistance, contact CME@medscape.net. American Medical Association’s Physician’s Recognition Award (AMA PRA) credits are accepted in the US as evidence of participation in CME activities. For further information on this award, please go to https://www.ama-assn.org. The AMA has determined that physicians not licensed in the US who participate in this CME activity are eligible for AMA PRA Category 1 Credits™. Through agreements that the AMA has made with agencies in some countries, AMA PRA credit may be acceptable as evidence of participation in CME activities. If you are not licensed in the US, please complete the questions online, print the AMA PRA CME credit certificate, and present it to your national medical association for review.

## Post-Test Questions

### Study Title: State-Specific Prevalence of Adult Tobacco Product Use and Cigarette Smoking Cessation Behaviors, United States, 2018–2019

#### CME Questions

1. Which of the following statements regarding current use of tobacco products in the United States in the study by Cornelius and colleagues is most accurate?
  - The median use nationwide of any tobacco product was 3.5%
  - The median rate of current cigarette smoking was 3.1%
  - The median rate of e-cigarette use was 2.4%
  - Water pipe use was more common than smokeless tobacco use
2. Current cigarette use was highest in what region of the United States in the current study?
  - The Rust Belt
  - Plains States
  - The South
  - Alaska and Hawaii
3. Which of the following statements regarding tobacco cessation behaviors in the current study is most accurate?
  - More than three-quarters of current cigarette smokers were interested in quitting
  - The region with the highest interest in quitting was the South
  - Almost 25% of adults who smoked had made an attempt to quit in the past year
  - Almost 15% of adults reported successfully quitting cigarettes in the past year
4. Which of the following statements regarding assistance in attempts to quit smoking cigarettes in the current study is most accurate?
  - Only half of smokers had received advice from a medical doctor to quit
  - The use of cessation counseling was approximately 40% during quit attempts
  - The use of medical therapy was approximately 30% during quit attempts
  - The highest use of cessation counseling plus medical therapy occurred in the Pacific region
